# Comparative Assessment of Hyperspectral Image Segmentation Algorithms for Fruit Defect Detection Under Different Illumination Conditions

**DOI:** 10.3390/jimaging12040160

**Published:** 2026-04-08

**Authors:** Anastasia Zolotukhina, Anton Sudarev, Georgiy Nesterov, Demid Khokhlov

**Affiliations:** Scientific and Technological Centre of Unique Instrumentation of the Russian Academy of Sciences, 15 Butlerova, 117342 Moscow, Russia

**Keywords:** spectral imaging, illumination, diffuse reflectance, fruit defect detection, machine learning, image segmentation

## Abstract

This study presents a comparative analysis of hyperspectral image segmentation algorithms for fruit defect detection under different illumination conditions. The research evaluates the performance of four segmentation methods (Spectral Angle Mapper, Random Forest, Support Vector Machine, and Neural Network) using three distinct illumination modes (local, simultaneous and sequential). The experimental setup employed hyperspectral imaging to assess tomato fruit samples, with data acquisition performed across the 450–850 nm spectral range. Quantitative metrics, including accuracy, error rate, precision, recall, F1-score, and Intersection over Union (IoU), were used to evaluate algorithm performance. Key findings indicate that Random Forest demonstrated superior performance across most metrics, particularly under simultaneous illumination conditions. The highest accuracy was achieved by Random Forest under sequential illumination (0.9971), while the best combination of segmentation metrics was obtained under simultaneous illumination, with an F1-score of 0.8996 and an IoU of 0.8176. The Neural Network showed competitive results. The Spectral Angle Mapper proved sensitive to illumination variations but excelled in specific scenarios requiring minimal memory usage. By demonstrating that acquisition protocol optimization can substantially improve segmentation performance, our results support the development of accurate, non-contact, high-throughput inspection systems and contribute to reducing postharvest losses and improving supply chain quality control.

## 1. Introduction

Spectral imaging techniques, including multispectral imaging and hyperspectral imaging (HSI), demonstrate significant potential for non-contact optical measurement of various physical and chemical properties. These techniques find extensive application in remote sensing [1,2,3], agricultural monitoring [4,5,6], medical diagnostics [7,8,9], nondestructive evaluation and quality control [9,10,11]. Many spectral imaging techniques rely on measurements of reflected light spectra [3], including those used for fruit defect detection.

Spectral imaging-based fruit defect detection is widely adopted for its capacity to enable non-destructive, rapid fruit inspection under diverse conditions—including field and greenhouse environments [12], as well as throughout the supply chain from farm to end consumer [13]. Increasingly, spectral imaging techniques are integrated with machine vision methods [14,15,16]. A diverse array of approaches is employed [17,18], with simplicity and accuracy often serving as key criteria for method selection. However, the growing number of available methods makes selecting the most suitable one increasingly important. This also highlights the need for studies that compare multiple methods for a specific task and a specific object of interest.

At the same time, spectral data acquisition typically involves multiple illumination modes. In field-based imaging scenarios, natural sunlight produces distinct illumination patterns: a local mode on sunny days and a quasi-uniform mode under cloudy conditions. In greenhouse environments and automated fruit sorting systems, artificial illumination setups generally provide multi-point configurations, either simultaneous quasi-uniform or sequential illumination patterns.

The shape of the studied object can also affect imaging results. Fruits are generally non-planar, which can lead to variations in signal intensity unrelated to differences in biochemical composition. As a result, a segmentation algorithm may misclassify the fruit edges or specular highlights as defects. Researchers address this problem in various ways. One is to optimize the illumination setup (e.g., using multiple lamps) and other imaging components (such as a light box or object-rotation mechanisms) to achieve the most uniform illumination over as much of the fruit surface as possible [19,20,21]. Another approach is to optimize the acquisition protocol to isolate the diffuse component of the object’s reflectance [22]. However, we have not found studies that compare the effectiveness of these approaches across different spectral image processing and analysis algorithms.

Given the variety of available techniques, it was necessary to determine the most effective spectral imaging approach for automated detection of fruit defects under different imaging modalities. Consequently, this study undertakes a comparative analysis to address this challenge. We designed an experimental setup capable of delivering three distinct illumination modes: local, simultaneous, and sequential. For each illumination mode we applied four widely used algorithms to identical datasets. Tomato fruit was selected as the primary object of study because tomatoes are among the fruit types most sensitive to mechanical damage [23]. Tomatoes are produced at a large scale and are highly perishable [24], making the development of automated, non-contact, high-throughput inspection methods particularly important. In addition, similar geometric challenges (surface curvature, glossiness, and localized skin defects) are common across many fruits and vegetables, including apples, pears, plums, cherries, nectarines, citrus fruits, peppers, and eggplants.

## 2. Materials and Methods

### 2.1. Sample Preparation

As part of this research, a controlled storage experiment was conducted. The samples were kept under typical short-term storage conditions: at 20 °C, away from direct sunlight, in a ventilated room. On the initial day of the experiment, 24 tomatoes of the “Pink Champion” variety were selected. The average fruit diameter was 80 mm. Each tomato was individually wrapped in paper and stored in a dark room. Tomatoes were removed daily for image acquisition. Image acquisition was performed daily for 5 consecutive days, with all measurements conducted at a consistent time of day to minimize diurnal variability.

Half of the samples exhibited defects (mechanical damage and developing rot), while the other half were healthy. Ground-truth information on the presence and extent of defects was obtained by an expert, who annotated the RGB images of the fruits. This resulted in pixel-wise labels, with each pixel assigned to one of three categories: background, healthy fruit tissue, or defect region.

### 2.2. Hyperspectral Imaging Setup and Illumination Modes

**Figure 1:**
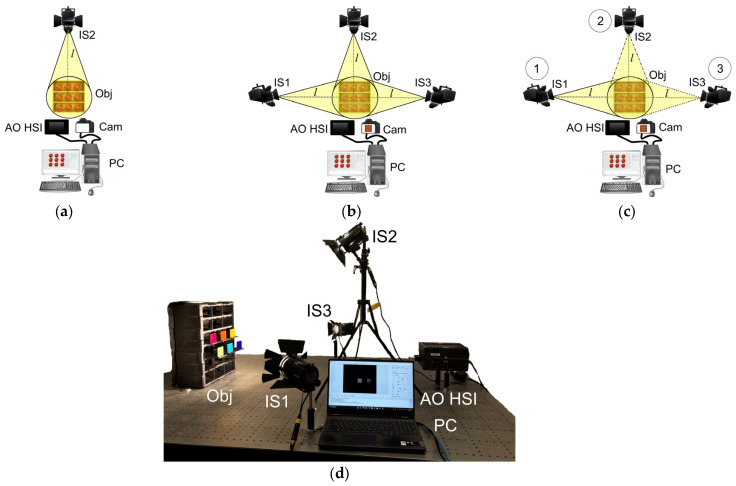
Local (**a**), simultaneous (**b**), sequential (**c**) illumination acquisition modes and experimental setup (**d**). AO HSI—acousto-optical hyperspectral imager; IS1, IS2, IS3—halogen lamps; Obj—object; Cam—color camera; PC—personal computer.

This study involved three methods for acquiring hyperspectral data, each based on distinct illumination schemes. Local illumination method employed a single-position illumination setup with a zenith angle of 45 degrees, as illustrated in Figure 1a. Simultaneous illumination method aimed to simulate uniform illumination conditions. This was achieved using three light sources positioned at an identical zenith angle but differing radial positions (Figure 1b). The sequential illumination method focused on isolating the volume component while eliminating specular reflection contributions. This method involved capturing a series of images with sequentially activated light sources at different positions (fixed zenith angle), as shown in Figure 1c. The spectral radiance of backscattered radiation was then determined by solving a system of linear equations [22].

For the local illumination mode, the 0/45 geometry was used, consistent with the standard object-color measurement geometries recommended by the International Commission on Illumination, whereas the simultaneous and sequential illumination modes were considered experimental configurations introduced to evaluate the influence of the illumination arrangement on segmentation performance.

A comparative evaluation of the methods’ effectiveness was conducted using an experimental setup, the schematic of which is presented in Figure 1d. The system comprised three identical halogen lamps (DLH4, Dedo Weigert Film GmbH, Munich, Germany) with a color temperature of 3000 K, CRI around 100, for target illumination and an acousto-optical hyperspectral imager (AO HSI), (Field HSI P1, STC UI RAS, Moscow, Russia) to record scattered radiation. Hyperspectral imaging was performed across a spectral range of 450–850 nm. Typical data acquisition parameters included: spectral resolution 5 nm, exposure time 500 μs and gain factor 20. The color camera (DFK 33UX250, The Imaging Source, Bremen, Germany) captured additional images. The reference spectrum is collected from the reflection of halogen lamps off a specific reference plate placed in object stand. The reference plate is made of a fluoropolymer with a reflection coefficient close to 1 across the entire operating spectral range of the AO HSI.

In the local illumination mode, the illuminance in the object plane at the center of the illuminated field was approximately 1.8 × 10^3^ lx, whereas in the simultaneous illumination mode it reached approximately 5.3 × 10^3^ lx, corresponding to one and three Dedolight DLH4 lamps, respectively, directed toward the same area.

### 2.3. Data Preprocessing

The input data for processing in the three operating modes of the system consisted of spectral image sets of the objects, $I_{obj,i}\left(x,y,\lambda \right)$ and the reference, $I_{ref,i}\left(x,y,\lambda \right)$, where *i* = [1, 2, 3] denotes the illumination angle. To suppress high-frequency detector noise, each spectral image was preprocessed using a two-dimensional median filter with a 3 × 3 kernel. For the local illumination mode, the spatial distribution of the reflection coefficient was estimated using the flat-field method [25], defined as follows:

(1) $$R^{local}\left(x,y,\lambda \right)=\frac{I_{obj,2}\left(x,y,\lambda \right)}{I_{ref,2}\left(x,y,\lambda \right)}.$$

The simultaneous mode was modeled by averaging the pixel-wise signals across all three illumination angles, thereby yielding the corresponding reflectance data:

(2) $$R^{simultaneous}\left(x,y,\lambda \right)=\frac{\sum_{i=1,2,3}I_{obj,i}\left(x,y,\lambda \right)}{\sum_{i=1,2,3}I_{ref,i}\left(x,y,\lambda \right)}.$$

We separated the volumetric and specular reflection components in the hyperspectral data under sequential illumination by following the algorithm [22]. After we computed the spatial distributions of the total reflection coefficient for each of the three local illumination angles, we applied an iterative pixel-wise procedure. Based on model (3), we performed a constrained nonlinear least-squares fitting using the lsqnonlin solver in MATLAB R2024b.

(3) $$R(x,y,\lambda )=R_{vol}(x,y,\lambda )+\varepsilon (x,y)\times R_{surf}(x,y,\lambda ).$$

This procedure yielded three parameter groups: the volume reflectance spectrum $R_{vol}(\lambda )$, the specular reflectance spectrum $R_{surf}(\lambda ),$ and the specular amplitude coefficients *ε* for each illumination angle. We constrained $R_{surf}(\lambda ),$ to the range [0, 0.1] and limited both $R_{vol}(\lambda )$ and *ε* to the interval [0, 1]. For initialization, we used the angle-averaged spectra as the starting point for the volume reflectance component, a constant value of 0.05 for the specular component, and angle-dependent estimates of the amplitude coefficients derived from the residuals between the measured data and the model. As a result, we obtained spatial maps of the volume reflectance component across the entire spectral range of interest.

**Figure 2:**
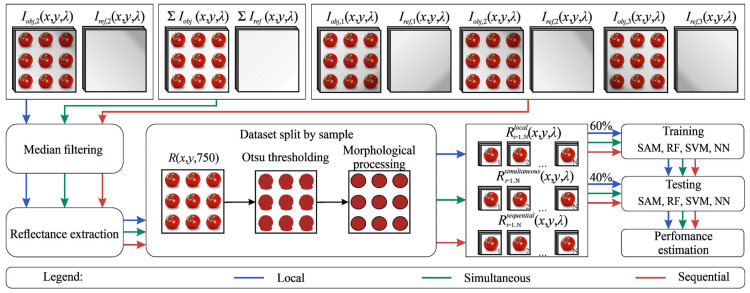
Preprocessing pipeline.

Next, we separated each dataset $R^{local}\left(x,y,\lambda \right)$, $R^{simultaneous}\left(x,y,\lambda \right)$ and $R^{sequential}\left(x,y,\lambda \right)$ containing six study objects in the field of view into individual datasets for each object using automated binary mask generation. To create the masks, we applied thresholding to the spectral image at 750 nm, a channel that provides high contrast between the objects and the background, using Otsu’s method. We then removed noisy regions outside the objects through morphological filtering, keeping only areas containing between 20,000 and 40,000 pixels, which roughly corresponded to the frame area occupied by each object. It should be noted that the hyperspectral data were acquired as a single image containing all 24 tomatoes simultaneously within the field of view, and individual hyperspectral cubes corresponding to each tomato were extracted only during preprocessing. As a result, we obtained three datasets for each imaging mode, each consisting of 24 hyperspectral cubes. We also generated these datasets for each of the five days of the experiment. This preprocessing pipeline is shown in Figure 2.

We randomly split the resulting datasets, each containing 120 spectral cubes, into training and testing sets in a 40/60 percent ratio. Each cube was manually labeled by a specialist based on RGB images of each sample, which were captured simultaneously with the hyperspectral data, to support subsequent algorithm training and performance evaluation. The same preprocessed spectral datasets were used for all evaluated methods within each illumination mode.

### 2.4. Segmentation and Classification Methods

We conducted defect classification on the processed datasets using four of the most common algorithms: Spectral Angle Mapper (SAM), Random Forest (RF), Support Vector Machine (SVM) and Neural Network (NN). For all illumination modes, the same model hyperparameters were used for each method. To reduce the effect of class imbalance during training, random under-sampling of the healthy class was applied in the training set so that the numbers of healthy and defective spectra were equal.

We chose SAM to represent simple spectral classification methods because it calculates the angle in n-dimensional space between the test spectrum and a reference class spectrum [26]. For the SAM analysis, 40% of the data were randomly selected for training to establish reference spectra for both defective and healthy regions. The split was performed at the level of individual fruits, with the subsets formed so as to minimize differences in the proportion of defective and healthy spectra between the training and test sets. The algorithm was then applied to the remaining 60% of the data. For each pixel, we calculated the dot product of the normalized pixel spectrum and the normalized reference spectrum to determine the cosine of the angle between them. This process produced spectral angle maps for each pixel, indicating their similarity to the defective and healthy classes. Finally, we created defect masks by applying Otsu’s method to threshold the similarity map of the class with the highest contrast.

We selected RF due to its popularity as a machine learning algorithm [27]. This algorithm constructs an ensemble of decision trees and evaluates each branch to classify the data effectively. For RF, we implemented an ensemble model in MATLAB using the TreeBagger function. We trained the model on a subset comprising 40 percent of the hyperspectral cubes, which were randomly selected. Each pixel served as a training object represented by its spectral data, while the target variables corresponded to the class labels for healthy and defective tissue. The ensemble was constructed with 100 decision trees, ensuring robust results while keeping computational costs manageable. The RF model was trained in classification mode with out-of-bag prediction enabled, and no additional hyperparameter tuning was performed. The model was used with the default classification settings, and we evaluated its quality using the out-of-bag prediction error.

For NN, we implemented a shallow 1-D convolutional NN that processes each pixel’s spectrum independently [28]. The model consists of three successive convolutional blocks with vertical kernels of size 5 and eight feature maps per block, each followed by batch normalization and ReLU. A global average-pooling layer collapses the spectral dimension into a single feature vector, which is passed through fully connected layers with 64 and 16 neurons (ReLU), and a final softmax output layer. We trained it with the Adam optimizer using an initial learning rate of 0.001, a mini-batch size of 4096.

For SVM, we used an RBF-kernel classifier, including automatic kernel scale selection, the SMO solver, a one-vs-one scheme for multiclass classification solver and an iteration limit of 10,000 [29].

### 2.5. Performance Metrics

We evaluated the results of automated classification using the following metrics:

(4) $$Accuracy=\frac{TP+TN}{TP+TN+FP+FN},$$

(5) $$ErrorRate=\frac{FP+FN}{TP+TN+FP+FN}\cdot 100\%,$$

(6) $$IoU=\frac{TP}{TP+FP+FN},$$

(7) $$Precision=\frac{TP}{TP+FP},$$

(8) $$Recall=\frac{TP}{TP+FN},$$

(9) $$Specificity=\frac{TN}{TN+FP},$$

(10) $$F1=\frac{2\cdot Precision\cdot Recall}{Precision+Recall},$$

where TP represents true positives, TN—true negatives, FP—false positives, and FN—false negatives.

This set of metrics allows for a comprehensive evaluation of the classification results. Accuracy and Error Rate provide an overall measure of classification correctness. Precision and Specificity indicate the accuracy of identifying positive and negative classes, while Recall reflects the completeness of defect detection. F1 combines both precision and recall into a single metric. Additionally, Intersection over Union (IoU) assesses the spatial agreement between predicted and true regions, which is particularly important for segmentation tasks [30].

## 3. Results

### 3.1. Spectral Validation of Experimental Setup

**Figure 3:**
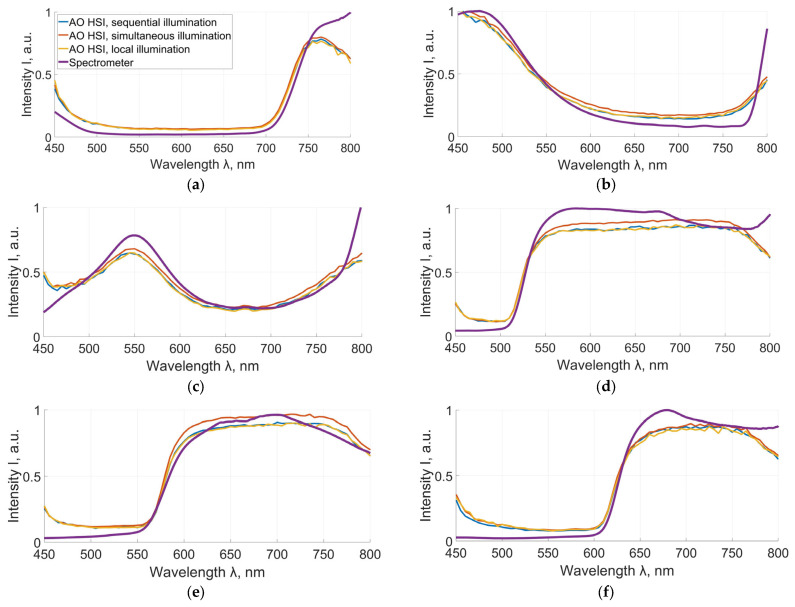
Spectra of violet (**a**), blue (**b**), green (**c**), yellow (**d**), orange (**e**) and red (**f**) test charts obtained by a spectrometer (violet line) and AO HSI in local (yellow line), simultaneous (red line) and sequential (blue line) illumination mode.

First, we validated the experimental setup under various illumination conditions. To accomplish this, six color standards were employed as test charts. These test charts were reference color plates representing different color shades: violet, blue, green, yellow, orange, and red. Their spectral signatures were acquired using both the experimental hyperspectral system and a reference diffraction spectrometer (Ocean Flame). Figure 3 presents the resulting spectral curves. Comparison showed that the mean absolute deviation from the reference spectrometer spectrum was about 0.08 for sequential illumination, 0.16 for simultaneous illumination, and 0.08 for local illumination, while the corresponding RMSE values were approximately 0.10 for all three illumination modes.

### 3.2. Segmentation Performance on Tomato Samples

**Figure 4:**
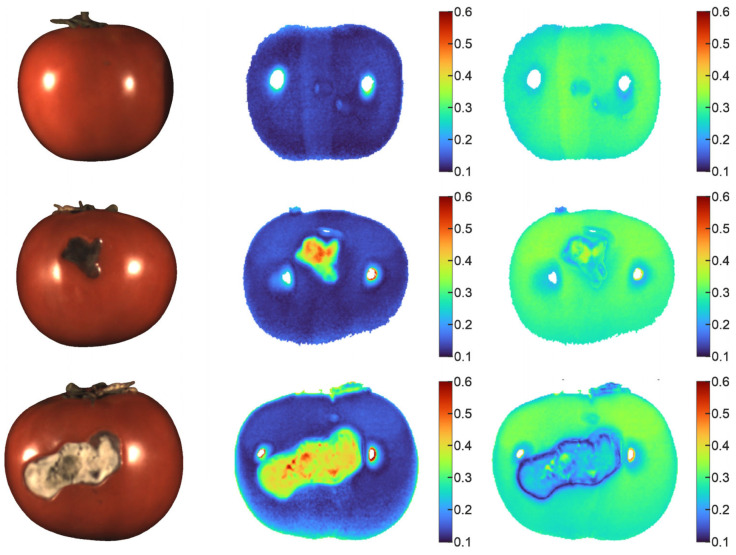
RGB images of several samples (**left column**) and spectral angle maps showing the correspondence of pixel spectra to the spectra of healthy regions (**center column**) and defective regions (**right column**). The color bar shows the spectral angle values in the spectral angle map (SAM), ranging from 0.1 in blue regions to 0.6 in red regions.

To optimize the SAM algorithm, we compared the contrast of spectral angle maps between the pixel spectra and the reference spectra for healthy and defective regions (Figure 4). In the analyzed images, the average size of one tomato was approximately 250 × 250 pixels. We selected the similarity map with the healthy region spectrum for threshold-based binarization using a threshold of 0.2. We also excluded glare areas from these maps in advance, as they do not carry information about the biochemical composition of the object and are fully characterized by the specular component. In subsequent analysis, we treated these areas as part of the healthy sample, since the appearance of skin defects, which is the primary focus of this study, causes the surface to lose turgor and prevents it from producing glare.

**Figure 5:**
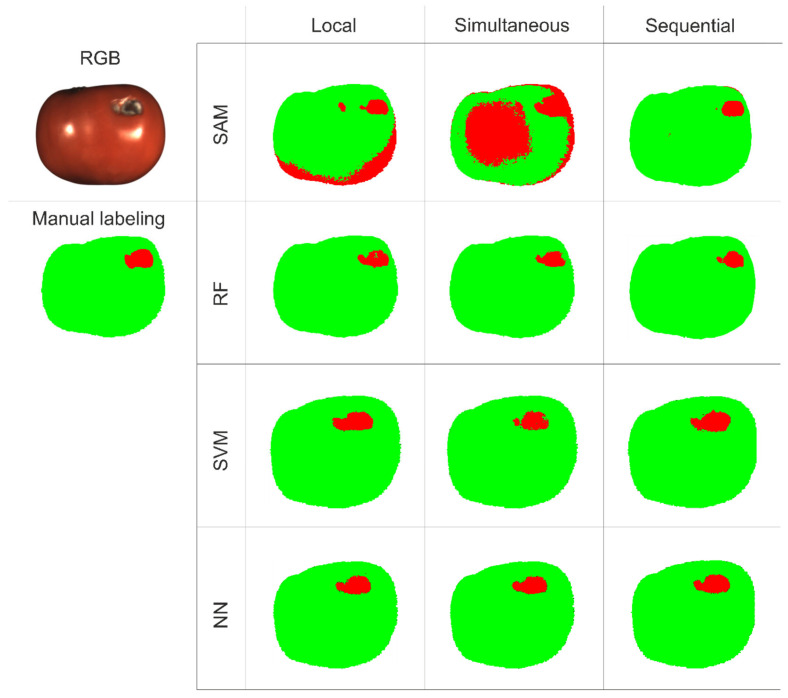
RGB image of a sample, manual annotation result, and decision maps for each combination of processing algorithm and illumination mode.

In Figure 5, we present the decision maps generated by the four algorithms for the three data acquisition modes: local illumination, simultaneous and sequential illumination from three angles, which are used for extraction of the diffuse reflection component.

**Figure 6:**
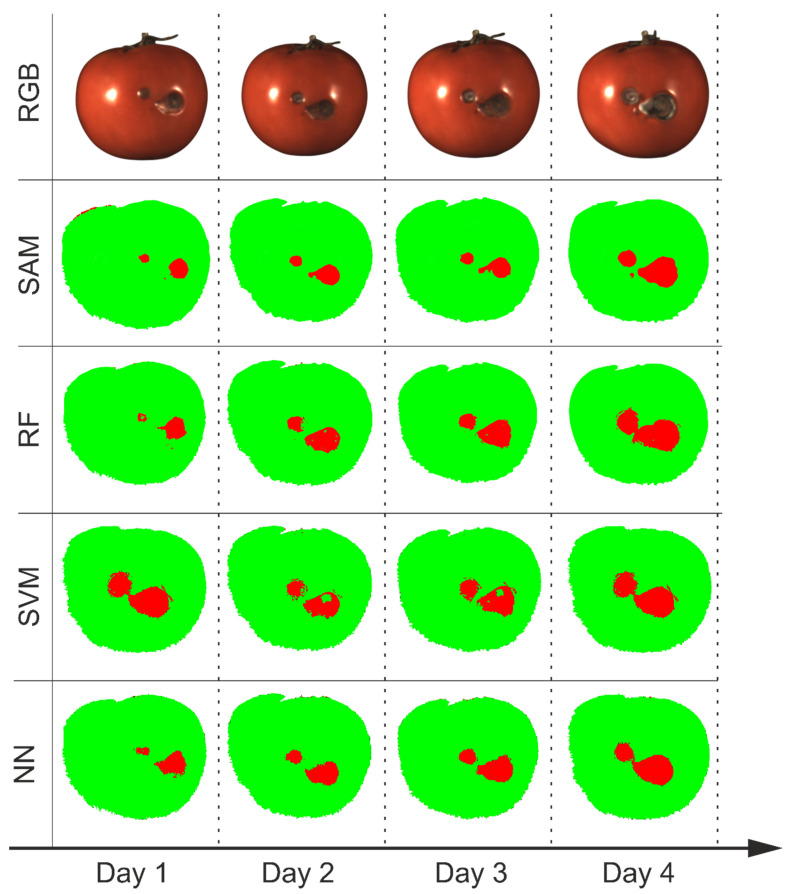
RGB images and decision maps obtained by the algorithms for the sequential illumination mode.

The decision maps show that the NN, SVM and RF algorithms demonstrated significantly greater robustness to changes in illumination mode. However, for the SAM algorithm, the highest agreement with the manual annotations was achieved under sequential illumination. Overall, SAM proved highly sensitive to the illumination conditions and tended to classify regions of uneven lighting, such as shadows in the local mode and central brightness variations in the simultaneous mode, as defects. Figure 6 illustrates the tracking of defect development on the same sample over four days of observation using all algorithms on the diffuse reflection data. All algorithms except SVM confirmed the visually observed growth of the defective area in this mode. Notably, SAM and NN more accurately highlighted a minor defect on the left side on the first day. It is also worth noting that RF and SVM require significantly more memory to store the model than NN and SAM.

### 3.3. Quantitative Performance Metrics

We evaluated the performance metrics for various illumination modes and processing algorithms on 72 samples not included in the training set, as summarized in Table 1 and Table 2. Accuracy was highest when extracting the volume reflectance component under sequential illumination. However, high values were also noted in other illumination modes due to the imbalanced training set (27,956 defective vs. 136,267 healthy pixels). This trend was consistent for the Error Rate. The Precision metric indicated that the SAM algorithm was twice as likely as RF to classify healthy pixels as defective, but its errors significantly decreased under sequential illumination. RF demonstrated high precision across all modes, peaking under simultaneous illumination. This pattern was mirrored in the F1 metric, which combines Precision and Recall. Recall was highest for the SAM and simultaneous illumination combination. At the same time, Specificity showed RF models excelled in correctly identifying healthy pixels across all modes, with SAM performing well under sequential illumination. The IoU metric revealed the best overlap with expert annotations for SAM under sequential illumination and for RF under simultaneous illumination. When we compared different metrics for SVM and the NN, we could not give a clear recommendation on the best lighting type, because different metrics preferred different lighting regimes, and the results were mixed. However, RF achieved the highest accuracy of all methods under simultaneous illumination.

## 4. Discussion

The NN algorithm achieved high accuracy for tomato defect detection. This agrees with prior studies [13,31] reporting accuracies of 0.96 under local illumination and 0.98 under simultaneous illumination. In study [23] on a machine-vision system for tomato sorting, SVM performed best among the tested methods. Under simultaneous illumination, SVM achieved an accuracy of 0.97. In the same setup, NN reached 0.96, and RF—0.94. SAM is used for fruit defect detection [32,33,34], and reported accuracies range from 0.75 to 0.99. However, studies on tomatoes mainly rely on three other algorithms.

The processing methods differ markedly in their performance characteristics. SAM is not suitable for precise segmentation due to a high number of false positives and extremely low F1 and IoU scores. It can only be used in applications where it is more important not to miss any defects, as indicated by high Recall, but the overall quality of segmentation suffers. This approach is relevant when it is critical to detect every defect, even at the cost of reduced annotation accuracy, although the issue may be partly related to threshold settings. The maps in Figure 4 are of interest for further analysis and the development of an adaptive threshold that reflects the degree of spoilage or damage to the produce. Such an evaluation is more biochemically meaningful because hyperspectral data capture not only visual features but also tissue composition changes associated with decay, mold, or mechanical damage. In this context, SAM can serve as a preliminary analysis tool, highlighting potential risk areas based on spectral deviations, even if spatial localization accuracy is low.

In this study, RF significantly outperformed other methods across most metrics. The best balance between precision and Recall was achieved with the simultaneous illumination mode, yielding an F1 score of 0.8996, an IoU of 0.8176, a Precision of 0.9522, and a Recall of 0.8525. Sequential illumination also showed high performance and can be considered an alternative when creating more uniform multi-point illumination is technically or economically challenging. Of particular interest are the high Accuracy (greater than 0.99) and Specificity (approximately 0.999), which virtually eliminate the misclassification of healthy produce as defective. This is critically important for industrial sorting lines, where false rejection directly results in financial losses. However, this model requires a substantial amount of memory for storage.

The remaining methods, SVM and NN, generally outperformed the SAM in quantitative performance metrics and yielded comparable results across all three illumination modes. Moreover, they also achieved the lowest Error Rate according to this metric. However, as illustrated in Figure 6, it is notable that SVM was the only method demonstrating minimal sensitivity to the temporal progression of defects. Particularly notable are the high Accuracy values (greater than 0.99) and Specificity (approximately 0.999), which virtually eliminate the misclassification of healthy produce as defective. This is critically important for industrial sorting lines, where false rejection directly results in financial losses.

The central practical question is which method is most appropriate under different illumination conditions. Based on our analysis, RF emerges as the optimal choice under conditions simulating overcast days in field conditions or intentionally implemented quasi uniform, or a simultaneous illumination. In contrast, NN is recommended for scenarios of all three illumination modes. SAM retains a narrow niche for preliminary analysis in resource-constrained environments where minimal memory usage is required, for example, because the model sizes were 35 KB for SAM, 200 KB for NN, 26 MB for SVM, and 1 GB for RF.

## 5. Conclusions

In summary, we conducted a study to evaluate algorithm performance metrics under three primary illumination conditions, using tomato fruit as a test case. The evaluation identified two most suitable algorithms: RF, a more complex method that delivered the highest performance, achieving the highest accuracy of 0.9971 under sequential illumination and the best balance of segmentation metrics under simultaneous illumination (F1 = 0.8996, IoU = 0.8176); and convolutional NN, a simpler approach with marginally lower, yet still competitive, results (accuracy = 0.9952–0.9962, F1 = 0.8356–0.8625). The results highlight that both factors (the acquisition protocol and the segmentation algorithm) affect the final performance. In applications where using complex algorithms or advanced hardware is not feasible, sufficiently high accuracy can still be achieved by adjusting the acquisition protocol. Separating the specular and diffuse components has been applied to fruit sorting for the first time to the best of our knowledge, resulting in a substantial improvement in defect-detection accuracy for the SAM method, whose F1-score increased from 0.1572 to 0.1666 to 0.5785 under sequential illumination.

The scientific novelty of this study lies in the application of a volumetric reflectance coefficient recovery method, originally developed for medical applications, to fruit defect detection. The study leads to several practical recommendations. Random Forest combined with simultaneous illumination should be preferred when the main objective is maximum segmentation accuracy. The neural network may be selected when stable performance across multiple illumination scenarios is required. Sequential illumination with separation of diffuse and specular components is advisable when lightweight spectral methods such as SAM are used and hardware or computational resources are limited.

The study addresses common geometric and illumination-related challenges and may serve as a basis for further research on fruits with different surface properties, including curved and glossy surfaces. We anticipate that these findings will be valuable for research groups and practitioners in agrotechnology and digital agriculture.

## Figures and Tables

**Table 1:** Performance metrics for the combination of illumination modes and SAM and RF algorithms. The best metric values obtained for the three illumination methods are shown in bold.

	SAM			RF		
	Local	Simultaneous	Sequential	Local	Simultaneous	Sequential
Accuracy	0.8544	0.8197	**0.9869**	0.9957	0.9965	**0.9971**
ErrorRate, %	14.56	18.03	**1.31**	0.43	0.35	**0.29**
F1	0.1666	0.1572	**0.5785**	0.8736	**0.8996**	0.8808
IoU	0.0909	0.0853	**0.4069**	0.7755	**0.8176**	0.7870
Precision	0.0931	0.0859	**0.4801**	0.9334	**0.9522**	0.9134
Recall	0.7957	**0.9185**	0.7275	0.8210	**0.8525**	0.8505
Specificity	0.8555	0.8179	**0.9901**	0.9989	**0.9992**	0.9990

**Table 2:** Performance metrics for combination of illumination modes and SVM and NN algorithms. The best metric values obtained for the three illumination methods are shown in bold.

	SVM			NN		
	Local	Simultaneous	Sequential	Local	Simultaneous	Sequential
Accuracy	0.9960	0.9957	**0.9966**	0.9953	0.9952	**0.9962**
ErrorRate, %	0.0040	0.0043	**0.0034**	0.0047	0.0048	**0.0038**
F1	**0.8842**	0.8734	0.8597	0.8616	**0.8625**	0.8356
IoU	**0.7924**	0.7753	0.7539	0.7568	**0.7582**	0.7176
Precision	0.9299	**0.9392**	0.8774	**0.9328**	0.9152	0.9012
Recall	**0.8428**	0.8162	0.8427	0.8004	**0.8155**	0.7788
Specificity	0.9988	**0.9990**	0.9985	**0.9989**	0.9986	**0.9989**

## Data Availability

Experimental data is available from the corresponding author upon reasonable request.

## Abbreviations

The following abbreviations are used in this manuscript:

AO HSI	Acousto-optical hyperspectral imager
NN	Neural network
RGB	Red–green–blue
RF	Random forest
SAM	Spectral angle mapper
SVM	Support vector machine
